# Supplementary material for: Everyday life in a Swedish nursing home during the COVID-19 pandemic: a qualitative interview study with persons 85 to 100 years
Source: BMJ Open. 2021 Jun 18;11(6):e048503. doi: 10.1136/bmjopen-2020-048503 (PMC8214988; doi:10.1136/bmjopen-2020-048503)
Supplement: Author's manuscript [file bmjopen-2020-048503.draft_revisions.pdf]

BMJ Open

BMJ Open is committed to open peer review. As part of this commitment we make the peer review history of every article we publish publicly available.

When an article is published we post the peer reviewers' comments and the authors' responses online. We also post the versions of the paper that were used during peer review. These are the versions that the peer review comments apply to.

The versions of the paper that follow are the versions that were submitted during the peer review process. They are not the versions of record or the final published versions. They should not be cited or distributed as the published version of this manuscript.

BMJ Open is an open access journal and the full, final, typeset and author-corrected version of record of the manuscript is available on our site with no access controls, subscription charges or pay-per-view fees (http://bmjopen.bmj.com).

If you have any questions on BMJ Open's open peer review process please email info.bmjopen@bmj.com

BMJ Open

Everyday life in a Swedish nursing home during the COVID-19 pandemic: a qualitative interview study with the oldest old

Journal:	*BMJ Open*
Manuscript ID	bmjopen-2020-048503
Article Type:	Original research
Date Submitted by the Author:	29-Dec-2020

Complete List of Authors:	Lood, Qarin; University of Gothenburg Sahlgrenska Academy, Institute of Neuroscience and Physiology, Department of Health and Rehabilitation, Sahlgrenska Academy, Centre for Ageing and Health - AgeCap; La Trobe University School of Nursing and Midwifery<br>Haak, Maria; Kristianstad University, Research Platform for Collaboration for Health, Faculty of Health Science; Lund University Faculty of Medicine, Department of Health Sciences<br>Dahlin-Ivanoff, Synneve; University of Gothenburg Sahlgrenska Academy, Institute of Neuroscience and Physiology, Department of Psychiatry and Neurochemistry, Sahlgrenska Academy, Centre for Ageing and Health - AgeCap

Keywords:	COVID-19, QUALITATIVE RESEARCH, PUBLIC HEALTH

SCHOLARONE™
Manuscripts

I, the Submitting Author has the right to grant and does grant on behalf of all authors of the Work (as defined in the below author licence), an exclusive licence and/or a non-exclusive licence for contributions from authors who are: i) UK Crown employees; ii) where BMJ has agreed a CC-BY licence shall apply, and/or iii) in accordance with the terms applicable for US Federal Government officers or employees acting as part of their official duties; on a worldwide, perpetual, irrevocable, royalty-free basis to BMJ Publishing Group Ltd ("BMJ") its licensees and where the relevant Journal is co-owned by BMJ to the co-owners of the Journal, to publish the Work in this journal and any other BMJ products and to exploit all rights, as set out in our licence.

The Submitting Author accepts and understands that any supply made under these terms is made by BMJ to the Submitting Author unless you are acting as an employee on behalf of your employer or a postgraduate student of an affiliated institution which is paying any applicable article publishing charge ("APC") for Open Access articles. Where the Submitting Author wishes to make the Work available on an Open Access basis (and intends to pay the relevant APC), the terms of reuse of such Open Access shall be governed by a Creative Commons licence – details of these licences and which Creative Commons licence will apply to this Work are set out in our licence referred to above.

Other than as permitted in any relevant BMJ Author's Self Archiving Policies, I confirm this Work has not been accepted for publication elsewhere, is not being considered for publication elsewhere and does not duplicate material already published. I confirm all authors consent to publication of this Work and authorise the granting of this licence.

For peer review only

Everyday life in a Swedish nursing home during the COVID-19 pandemic: a qualitative interview study with the oldest old

Qarin Lood[1,2] (0000-0002-6187-0929), Maria Haak[3,4] (0000-0002-0054-2542), Synneve Dahlin-Ivanoff[5] (0000-0002-3838-4527)

Qarin Lood associate professor Institute of Neuroscience and Physiology, Department of Health and Rehabilitation, Sahlgrenska Academy, Centre for Ageing and Health – AgeCap, University of Gothenburg. Maria Haak professor Research Platform for Collaboration for Health, Faculty of Health Science, Kristianstad University. Synneve Dahlin-Ivanoff professor Institute of Neuroscience and Physiology, Department of Psychiatry and Neurochemistry, Sahlgrenska Academy, Centre for Ageing and Health – AgeCap, University of Gothenburg.

Correspondence to Q Lood Qarin.lood@neuro.gu.se

1. Institute of Neuroscience and Physiology, Department of Health and Rehabilitation, Sahlgrenska Academy, Centre for Ageing and Health – AgeCap, University of Gothenburg, Gothenburg, Sweden
2. School of Nursing and Midwifery, La Trobe University, Melbourne, Australia
3. Research Platform for Collaboration for Health, Faculty of Health Science, Kristianstad University, Kristianstad, Sweden
4. Department of Health Sciences, Faculty of Medicine, Lund University, Lund, Sweden.
5. Institute of Neuroscience and Physiology, Department of Psychiatry and Neurochemistry, Sahlgrenska Academy, Centre for Ageing and Health – AgeCap, University of Gothenburg, Gothenburg, Sweden

For peer review only

Abstract
Objective
To understand and report on the impact of the COVID-19 pandemic on the everyday lives of frail older persons living in nursing homes by exploring their experiences of how the pandemic-related restrictions had influenced them and in what way.
Design
Empirical qualitative interview study.
Setting
A publicly run nursing home in Sweden in June 2020. The nursing home had been impacted by visitor restrictions, cancelled activities and physical distancing requirements since March 2020.
Participants
A random sample of 10 persons aged 85 to 100 years, recruited through nursing home management and interviewed using medically approved visors and physical distancing.
Analysis
The interviews were analysed using thematic analysis, which involves familiarisation, coding and definition of themes. Transcripts were coded into data-driven categories before being organised into categories that described and explained the data.
Results
The analysis resulted in the main theme It is like living in a bubble, and four sub-themes; Feeling taken care of, Living one day at a time, without fear of the virus, Being in the hands of others, and There is no icing on the cake. These findings visualise how pandemic-related restrictions in nursing homes represent a risk of isolating older people from the outside world, and diminishing their freedom.
Conclusions
Contributing to the growing area of COVID-19 related research, our study provides novel insights into the importance of balancing protection from SARS-CoV-2 and support of older persons' rights to decide for themselves how to spend the rest of their lives. Specifically, our study provides a foundation for future research on how to handle the transmission of SARS-Cov-2 without depriving older people of the things that makes life worth living.

Keywords: COVID-19, long-term care, social isolation, qualitative, older adults, healthy lives, well-being, sustainability

Article summary
Strengths and limitations of this study

- This is one of the first studies to explore the consequences of the ongoing pandemic from the perspective of older persons living in nursing homes, despite them being depicted as one of the most vulnerable groups in terms of severity of the disease
- The findings complement the medical understanding of the impact of COVID-19 on health and well-being on older persons' lives
- It was not possible to evaluate whether the pandemic presents an actual risk to the health of older persons in nursing homes, which represents a limitation to the study
- The number of participants was limited due to the restrictions applied and the participants were all living in the same nursing home which may raise questions to the representability of the findings
- Although representing a small Swedish example, the similarities between restrictions implemented in nursing homes around the world increases the international value of the study

For peer review only

Background

The novel Coronavirus SARS-CoV-2 that is causing COVID-19 has changed pretty much everything that people do. It spreads rapidly and could cause severe and fatal infections, especially among older persons living in nursing homes, who live with both physical frailty (1) and compromised physiological barriers (2). As per December 14 2020, a total of 10793 persons living in Swedish nursing homes had been confirmed with COVID-19, and 3447 (32%) of them had died with the disease. This constitutes 46% of the amount of people who had died with COVID-19 in Sweden at that time (7455 persons) (3), and persons in nursing homes are one of the most affected groups in terms of mortality globally. Out of fear of the virus, nursing home organisations around the world have therefore made drastic changes to their services to diminish the spread of infection, in line with international guidelines by the World Health Organization (4). This also applies in Swedish nursing homes that have implemented physical distancing among all people living and working in nursing homes, most organised activities are cancelled and strict visitor restrictions were imposed in all nursing homes from March 30 until October 1 2020. The visitor restrictions initiated a public debate on the risk of social isolation and the negative consequences that might have (5), and the restrictions were lifted in October 2020 based on the risk of negative consequences of isolation instigated by lengthy visitor restrictions (6). However, as a result of a drastic increase in transmission rates and COVID-related deaths in Sweden in November 2020, regional visitor restrictions were re-instated in December 2020, allowing nursing homes to ban visitors from entering the nursing home. Little is known on how this may impact the general health and well-being of persons living in nursing homes, since there is close to no primary data on their own experiences.

Already before the pandemic, there were reports on social isolation, limited quality of life and near endemic loneliness among persons living in nursing homes (7, 8). This poses serious threats to their everyday fulfilment and sense of dignity (9-11), and the pandemic tends to increase those threats due to the focus on reducing transmission. There are indications that the restrictive measures taken to protect people residing in nursing homes from COVID-19 may have a negative impact on their well-being, but there is little empirical support for this matter (12). What we do know, however, is that social isolation is a serious public health concern, with a negative impact on medical illness such as cardiovascular diseases (13), and mental health problems, such as loneliness, depression and cognitive decline (14). There are also recent reports on the negative impact of social disconnection and perceived isolation on both anxiety and depression among older persons (15).

According to the Swedish government (16), a modern society and welfare state needs to improve the possibilities for all older people to participate in society, to enjoy a decent quality of life, and to experience health, meaningful activities and social participation. Research on best practice nursing home care also puts focus on activities to promote thriving and support a good quality of life (17-19), and there is evidence to suggest that quality of life is improved by participating in personally meaningful activities (20). Yet, as part of one of the risk groups for severe disease or death from COVID-19, older persons living in nursing homes have faced restrictions that have resulted in no visitors, communal dining or leisure activities. The scientific community is responding rapidly to the consequences of those restrictions, but what has been left in the dark is how the persons in nursing homes themselves experience the measures taken to protect them. Therefore, this paper aimed to understand and report on everyday lives of frail older persons living in nursing homes by exploring their experiences of how restrictions related to the COVID-19 pandemic had influenced them and in what way.

Methods

Seeking to understand the complexity of everyday life in a nursing home during the COVID-19

For peer review only

pandemic, this study had a qualitative design to explore and describe older persons' experiences. The study involved individual interviews with 10 persons in a nursing home over a two-week period in June 2020. Ethical approval was obtained from The regional ethical board in Gothenburg (ref. no. 813-18).

Patient and public involvement

The primary focus of this study was to make the voices of frail older persons heard in relation research about and with them, to think differently, not only about research and care with frail older people, but also about older people, frailty and ageing more widely. Every effort was made to ensure that the choice to participate, and to what extent, was the person's own, not limited by research approaches or structures. The participants will also have the opportunity to take part of the study findings in a popular scientific report, or in dialogue with the first author depending on their choice of communication. As part of a larger research program on user involvement in research, the design of the study was discussed in seminars with user representatives from pensioner associations in Sweden before commencement. Drawing on the findings from this study, the user representatives will also be involved in future planning of research on user involvement in research on ageing and health, together with researchers and healthcare representatives from different disciplines and professions.

Study setting

Participants were recruited from a nursing home with 101 beds (out of which 27 were in dementia care units), and about 85 staff members. In Sweden, nursing homes is a living solution for persons 65 years of age and older, provided for persons with extensive care needs that cannot be attended to in ordinary housing. Care staff are available 24 hours, seven days a week, and registered nurses, physicians and allied health professionals are generally available for care and rehabilitation when needed. During the COVID-19 pandemic, however, such visits were confined to acute needs. In the nursing home included in this study, a total of four of the persons living there had contracted the virus by the time of data collection. All of them survived and were well at the time of data collection (two of them were included in the study). A few staff members had also contracted the virus and had been, or were, on sick-leave. Only outdoor visits from friends and family were allowed during the study period, with a maximum of two visitors at a time. Dates and times for visits were organised by nursing home managers, and the persons living in the nursing home had to have care professionals helping them back and forth to the outdoor area. Indoor visits were allowed for special reasons only, such as end-of-life-care or severe anxiety. Delivery of food or things from outside the nursing home had to be delivered through the care professionals. All care professionals were entitled to adhere to the recommendations by the Public Health Agency of Sweden; they were strongly encouraged not go to work when feeling ill, even with mild symptoms, and to follow basal hygiene routines such as hand hygiene and disinfection of areas in contact with human beings.

Participants and data collection

A random sample of 11 persons were invited to participate in the study through the nursing home managers in June 2020. Nursing home professionals made initial contact with people who fulfilled the criterion; being able to hold a conversation in Swedish for at least 15 minutes. One person withdrew their consent after the interview had been conducted, resulting in a total of 10 interviews since no more eligible persons were willing to participate. All data collected from the person who withdrew their consent were deleted and not included in the study, and no other person than the researcher who conducted the interview took part of the interview. The participants were between 85 and 100 years of age (85, 86, 89, 90, 94, 96, 98, 100, 100, and 100 years respectively), they were physically frail (21) and in need of at least one other person in activities of daily living. The first author conducted six interviews and the last author conducted

For peer review only

four interviews. Both interviewers were registered occupational therapists with previous experience of working with, and doing research with, frail older persons. The interviews were guided by an interview guide that focused on how the older persons' everyday life had been influenced by the pandemic and their increasingly frail bodies, and they lasted between 17 and 60 minutes. Seven participants were women, eight out of ten had children and three of them had partners who were still alive. Medically approved visors were used by the researchers in all contacts between them and the participants, along with physical distancing of at least two meters between the interviewee and the interviewer. All interviews were recorded digitally and transcribed by the first and second authors before analysis.

Data analysis

Data were analysed by all three authors who all had experience in doing qualitative research on ageing and health with and for frail older persons. using the step-wise procedure for thematic analysis (22) as follows. First, all authors listened to the interviews repeatedly to become familiarised with the data. Second, initial codes that described the participants' experiences in a condensed way were generated and discussed among the authors. Third, the initial codes were revised to search for themes, which involved a process of listening to and reading all collected data again. Notes were taken on essential meanings to extract data, which were interpreted and organized into a prospective thematic structure. Fourth, the prospective themes were reviewed and triangulated by the authors to define the relationships between them and assess the validity of each prospective theme in relation to the data set as a whole. Fifth, data extracts within each identified theme were interpreted to define final themes based on the meaning and implications of the theme. Sixth, the scientific report was written. In the report, the themes are supported by quotations from the participants, and fictional names are used to put the quotations into context.

Results

It is like living in a bubble

Everyday life in a nursing home to the persons living there during the COVID-19 pandemic meant living a safe but limited life, interpreted as living in a bubble that represented both protection and isolation from the outside world. The nursing home was experienced as being somewhat a world of its own, separated from what was perceived as normal. Unlike what was perceived as being the situation for the rest of society, many aspects of everyday life could continue just like before the pandemic, and there were no experiences of an immediate threat from the virus. However, everyday life circled around the few opportunities to think and talk about other things than the pandemic, and the freedom to choose for oneself what to do, with whom and when were diminished. These different aspects of everyday life in the nursing home bubble are described in the four sub-themes: *Feeling taken care of, Living one day at a time without fear of the virus, Being in the hands of others,* and *There is no icing on the cake.*

Feeling taken care of

Living in a nursing home contributed to a feeling that one was taken care of, both in terms of being protected from the virus and being cared for by staff. Daily routines could still be carried out, representing an important part of everyday life that was not affected by the pandemic, and the nursing home staff embodied a much-appreciated human contact in times of social isolation. The nursing home staff also offered company and a sense of security in knowing that there would be someone there to care for you and provide support when needed. This made it possible to defy both the pandemic and the ageing body, providing support to maintain essential activities and daily routines, which contributed to an everyday life that, in many ways, were the same as before the pandemic. This is visualized by a quotation by Bertil's response to the question about how he felt about living in the nursing home during the pandemic:

For peer review only

'I am very satisfied. Because of the staff, that they are so helpful. They are helpful and come in and talk with me. I have nothing to complain about… I cannot manage on my own, here I am spoiled, here I get food and the assistance that I need.'

Living one day at a time, without fear of the virus

Living one day at a time, life in the bubble represented by the nursing home during the pandemic involved not worrying about tomorrow or fearing the virus. Even if the ageing and physically frail body represented an insecurity as to whether changes to daily routines would be possible to handle, it also represented an opportunity to seize the day because of the uncertainty of not knowing when life would end, regardless of the pandemic. Approaching the end of life, the participants experienced that they could choose to focus on, and appreciate, the small things in life and live one day at a time, rather than worrying for what might happen in the future. When being asked if she was afraid of the virus, Margaret replied:

'There is no need to worry. I live one day at a time, because I am so old.'

Being in the hands of others

The limitations of living in the shielded world of a nursing home during the pandemic involved not having the freedom to move about, to go outside or to receive visitors without assistance from staff. Although feeling taken care of, the limited freedom meant being in the hands of nursing home staff and authorities responsible for the restrictions. Instructions from nursing home staff and authorities were dutifully followed, even though it was sometimes difficult to understand why they were as they were. There was a perception that there simply was no option, and there were expectations on authority representatives to set a date for when the isolation would be over. The feeling of being in the hands of others is illustrated by Lisbeth's answer to the question: What is the biggest difference for you since the pandemic started?

'The freedom. I am dependent you see. So, I cannot choose... Nobody is allowed to go out, nobody is allowed to come in.'

There is no icing on the cake

As a result of the pandemic-related restrictions, everyday life in the nursing home came to circle around daily routines and news on the pandemic, and there were few or no opportunities to do what really mattered. The nursing home staff were perceived as being under extra pressure during the pandemic and there were doubts whether they could be expected to provide support in activities that were considered to be the icing on the cake. The days were perceived as empty and dull, and the telephone became a lifeline that contributed to a sense of normality and breath of fresh air in everyday life. Providing opportunities to think and talk about other things than the consequences of the pandemic, the telephone provided a familiar form of communication with people outside the nursing home, and it made it possible to dream about life after the pandemic, when meaningful activities and roles could resume and life could go back to normal. There was a strong wish for the restrictions to be over, and when being asked about the restrictions, Ebba said:

'I do not understand why a healthy person cannot come and visit, and help out, and why we need to sit two-and-two by the table. Then we cannot talk, so they do destroy life for you.'

Discussion

Statement of principal findings

To the best of our knowledge, this study is the first to explore experiences of everyday life in a nursing home during the COVID-19 pandemic. An important finding is the illumination of how

For peer review only

the restrictions implemented in nursing homes present serious challenges to older people's well-being and freedom of choice, by limiting their opportunities to do what they have a reason to value. Giving voice to the seldom heard group older persons living in nursing homes, the present findings contrast the idea of protection from SARS-CoV-2 as the most important health intervention in nursing homes, providing insights into how pandemic-related restrictions may have a more serious impact upon the health and well-being of persons living in nursing homes than the virus per se. Although not expressing a willingness to die, the participants were not afraid of the virus or what might happen should they contract it, and they wished for the restrictions to be over as soon as possible.

Strengths and weaknesses of the study

The major strength of this study is the uniqueness of the qualitative data gathered from a sample of persons 85 years and older living in nursing homes. This allowed us to explore their experiences of how the pandemic-related restrictions had influenced them and report in what way. However, potential effects of the pandemic on the health of the older persons could not be evaluated. A qualitative design was applied to provide first-hand understandings into the shielded world of nursing homes during the pandemic, and the number of participants was limited due to the restrictions applied. As described by Malterud (23), rigour in qualitative studies depends on reflexivity and not a specified sample size (23) and the breadth and uniqueness of the data provide important insight to the field of nursing home care in the light of the pandemic. What remains unknown is, however, whether the findings are a true representation. The findings complement the medical understanding of the impact of COVID-19 on health and wellbeing on older persons' lives, and although representing a Swedish example, the similarities between restrictions implemented in nursing homes around the world increases the international value of the study. Another question mark is whether the pandemic presents an actual risk to the health of older persons in nursing homes A suggestion for future research is, therefore, to evaluate the effects of protective models that consider the threats to both physical and mental health imposed by the pandemic internationally. Moreover, studying experiences the pandemic, while at the same time experiencing it could have resulted in a biased analysis. The methodological measures taken to prevent this was to include all authors, with different individual backgrounds, in the analysis process, striving to be aware of our own expectations and experiences. Every attempt has been made to assure that the authors' experiences did not overshadow the voices of the older persons participating in the study.

Other studies

Interpreted in relation to previous research on autonomy for older people in nursing homes (24), the present findings visualise a risk for measures taken to protect persons in nursing homes from COVID-19 to become paternalistic. Authorities and clinicians alike may believe that they know best, and older persons' interests and experienced needs may be overridden by others. Supported by what has been described by Dichter et al. (25), the findings call for a balance between infection control and person-centred care that involves persons on both ends of the care process. Combining a biomedical understanding of persons in need of care with a personal understanding of desires and needs, person-centred care has been described in relation to healthcare professionals' responsibility to meet each person's needs by personalising the care and prioritise shared decision-making (26). The COVID-19 pandemic has challenged this endeavour, and highlighted a need for infection control interventions to also provide psychosocial and mental health support (27).

The older persons' descriptions of everyday life in a nursing home during the COVID-19 pandemic illuminate weaknesses in terms of caring for older people as persons, and there is a need to find novel ideas for nursing homes to support older persons to both remain safe and

For peer review only

preserve their dignity and personhood. Providing empirical support for what until now has been assumptions based on the views of other people than the older persons themselves (12, 25), the present findings contribute with a unique insight into how pandemic-related restrictions in nursing homes may be experienced as isolation and deprivation of freedom of choice. Deepening the understanding of the negative consequences of the pandemic on older persons' everyday lives, the present findings thus provide a foundation for future research to attend to health and well-being in a broader sense than strict infection control. Hitherto, visitor restrictions are being implemented without scientific support for the effect of such measures to minimise virus transmission (28), and there is a lack on primary data on how this may impact the lives of persons living in, and visiting, nursing homes. This study thus presents a unique contribution to the growing area of research on the COVID-19 pandemic, visualising consequences of the pandemic-related restrictions through the metaphor of living in a bubble. Everyday life at the nursing home during the COVID-19 pandemic meant living a protected but isolated life and the present findings highlight the need for nursing homes to both aim to protect and sustain physical health and capacity, and to support psychosocial aspects of life. This correlates with pre-pandemic research on problems with loneliness and boredom in nursing homes (29), and supports the idea of the need for nursing homes to be facilitators of both physical and psychosocial health and well-being. As described by Landry et al. (30), public health measures to reduce transmission of COVID-19 among older persons need to attend to several dimensions of health (30), and contradictions between protection from the threats of COVID-19 infection and the risk of social isolation imposed by the pandemic needs to be attended to (12, 25, 30, 31). What the present study adds to the understanding of the impact of the COVID-19 pandemic is the perspective of representatives from a group identified as being among the most vulnerable when it comes to COVID-19 infection. Contrary to expectations, the findings illustrate how the older persons maintained many daily routines, without fear for the virus, and that they avoided pandemic worry by living one day at a time. This has been described in previous research as a strategy applied by persons approaching the end of life (32), and could be understood as a potential for health. In the light of these findings, it seems reasonable to question whether the interventions to minimise the threat of COVID-19 in nursing homes are adequate, and whether mortality is a sufficient outcome to measure effects of such interventions. Considered in relation to what has previously been reported on the negative impact of social isolation on older persons' health (13-15), the present findings could thus be considered a foundation for future research on how to support older persons to decide for themselves how to spend the rest of their lives.

Giving voice to persons representing one of the most vulnerable groups in terms of COVID-19 infection, this study contributes with a unique perspective on the impact of the COVID-19 pandemic on public health. The findings provide novel insights into the importance of balancing protection from COVID-19 and support of older persons' rights to decide for themselves how to spend the rest of their lives. Specifically, the study contributes with the visualisation of the impact of the pandemic, and not the virus per se, on the older persons' lives. This provides a foundation for future research with regards to how to handle the transmission of COVID-19, without depriving older people of the things that makes life worth living. From the older persons' own perspective, restrictions to minimise risk ought to be balanced with freedom to decide for oneself how to spend the rest of one's life. Mortality may not be a sufficient outcome when evaluating the impact of infectious diseases on the health and well-being of older persons in nursing homes. A suggestion for future research is therefore to evaluate the effects of interventions that consider threats to both physical and mental health of older persons living in nursing homes

List of abbreviations

For peer review only

COVID-19 – Corona virus disease 2019
SARS-CoV-2 – Severe acute respiratory syndrome coronavirus 2

Declarations

Ethics approval and consent to participate

Ethical approval was obtained from The regional ethical board in Gothenburg (ref. no. 813-18). In accordance with this approval, all eligible persons received a letter stating the purpose and methods of the study, that participation was voluntary and that their contributions would be kept confidential and unidentifiable in all reporting of the findings. All participants also signed an informed consent form before the data collection started. One participant chose not to participate after the interview, and all data from that participant were excluded from the analysis and all personal data from that applicant has been destroyed.

Consent for publication

All participants gave permission to use their anonymised data for publication purposes.

Data sharing statement

The qualitative data generated and analysed as part of the current study are not publicly available due to the information provided to the participants when obtaining their informed consent, stating that all attempts would be made to maintain confidentiality. De-identified data are, however, available upon reasonable request to enable review, and will be stored for 10 years at the University of Gothenburg. All data are covered by the Public Access to Information and Secrecy act (offentlighets- och sekretesslagen) and a confidentiality assessment (sekretessprövning) will be performed at each individual request. Permission from University of Gothenburg, the Institute of Neuroscience and Physiology, has to be obtained before data can be accessed.

Competing interests

None declared

Funding

This study was funded by The Swedish Research Council for Health, Working Life and Welfare (FORTE, 2018–00904). The funding body was not involved in the design of the study, the collection, analysis and interpretation of the data, nor in writing the manuscript. All authors had access to the full data in the study and accept responsibility to submit for publication.

Contributorship statement

QL designed and directed the project and initiated contact with the management at the nursing home. She also did the literature search and drafted the manuscript. QL and SDI collected the data, and were responsible for data analysis with input from MH. SDI also contributed with intellectual input on the manuscript draft. MH was responsible for the first steps of the analysis and contributed with intellectual input to the final interpretations as well as the manuscript draft. All authors have reviewed and agreed to the draft and final version of the paper.

Acknowledgements

First and foremost, we would like to thank the persons participating in the study, sharing their experiences of their everyday lives during these challenging times. We would also like to thank the management at the nursing home for allowing us to conduct the interviews, and the care staff for assisting us with selecting participants and for their tireless commitment to providing

For peer review only

good care and support to the persons living in the nursing home. Finally, we thank The Swedish Research Council for Health, Working Life and Welfare (FORTE) who funded the study.

For peer review only

References

1. Kojima G. Prevalence of frailty in nursing homes: A systematic review and meta-analysis. Journal of the American Medical Directors Association. 2015;16:940-5.
2. Stone P. Integration of infection management and palliative care in nursing homes: an understudied issue. Research in Gerontological Nursing. 2017;10:199-204.
3. The National board on health and welfare (Socialstyrelsen). Statistics on COVID-19 (In Swedish): National Board on Health and Welfare; 2020 [Available from: https://www.socialstyrelsen.se/en/statistics-and-data/statistics/statistics-on-covid-19/.]
4. World Health Organization. Preventing and managing COVID-19 across long-term care services: Policy Brief Geneva: World Health Organization; 2020 [Available from: https://apps.who.int/iris/handle/10665/334020.]
5. Ludvigsson J. The first eight months of Sweden's COVID-19 strategy and the key actions and actors that were involved. Acta Paediatrica. 2020;00:1-13.
6. The Public health agency of Sweden (Folkhälsomyndigheten). Preventing transmission of infection when visiting nursing homes (In Swedish): The public health agency of Sweden; 2020 [Available from: https://www.folkhalsomyndigheten.se/smittskydd-beredskap/utbrott/aktuella-utbrott/covid-19/information-till-varden/personal-inom-aldreomsorg/forebygga-smittspridning-vid-besok-pa-aldreboenden/.]
7. Jakobsson U, Hallberg I. Loneliness, fear, and quality of life among elderly in Sweden: a gender perspective. Aging Clinical and Experimental Research. 2005;17(6):494-501.
8. Sundström G, Fransson E, Malmberg B, Davey A. Loneliness among older Europeans. European Journal of Ageing. 2009;6(4):267-75.
9. Edvardsson D, Petersson L, Sjögren K, Lindkvist M, Sandman P. Everyday activities for people with dementia in residential aged care: associations with person-centredness and quality of life. International journal of older people nursing. 2013;9(4):269-76.
10. O'Sullivan G, Hocking C. Positive ageing in residential care. New Zealand Journal of Occupational Therapy. 2006;53(1):17-26.
11. Vernooij-Dassen M, Faber M, Olde Rikkert M, Koopmans R, van Achterberg T, Bratt D, et al. Dementia care and labour market: the role of job satisfaction. Aging and Mental Health. 2009;13(3):383–90.
12. Gordon A, Goodman C, Achterberg W, Barker R, Burns E, Hanratty B, et al. Commentary: COVID in care homes - challenges and dilemmas in healthcare delivery. Age and Ageing. 2020;49(5):701-5.
13. Tomàs J, Pinazo-Hernandis S, Oliver A, Donio-Bellegarde M, Tomàs-Aguirre F. Loneliness and social support: Differential predictive power on depression and satisfaction in senior citizens. Journal of Community Psychology. 2019;47(5):1225-34.
14. Barbosa Neves B, Sanders A, Kokanović R. "It's the worst bloody feeling in the world": Experiences of loneliness and social isolation among older people living in care homes. Journal of Aging Studies. 2019;49:74-84.
15. Santini Z, Jose P, Cornwell E, Koyanagi A, Nielsen L, Hinrichsen C, et al. Social disconnectedness, perceived isolation, and symptoms of depression and anxiety among older Americans (NSHAP): a longitudinal mediation analysis. The Lancet Public Health. 2020;5(1):E62-E70.
16. Government offices of Sweden (Regeringskansliet). Mål för socialtjänst inklusive äldreomsorg (In Swedisb) Government offices of Sweden; 2020 [Available from: https://www.regeringen.se/regeringens-politik/socialtjanst-inklusive-aldreomsorg/mal-for-socialtjanst-inklusive-aldreomsorg/.]
17. Bergland Å, Kirkevold M. Thriving in nursing homes in Norway: Contributing aspects described by residents. International journal of nursing studies. 2006;43(6):681-91.
18. Cooney A. Resident perspectives of the determinants of quality of life in residential care in Ireland. Journal of Advanced Nursing. 2009;65(5):1029-38.

For peer review only - http://bmjopen.bmj.com/site/about/guidelines.xhtml

19. Zingmark K, Sandman P, Norberg A. Promoting a good life among people with Alzheimer's disease. Journal of Advanced Nursing. 2002;38(1):50-8.
20. Cooney A, Murphy K, O'Shea E. Resident perspectives of the determinants of quality of life in residential care in Ireland. Journal of Advanced Nursing. 2009;65(5):1029-38.
21. Eklund K, Wilhelmson K, Landahl S, Dahlin-Ivanoff S. Screening for frailty among older emergency department visitors: Validation of the new FRESH-screening instrument. BMC Emergency Medicine. 2016;16(1):27.
22. Braun V, Clarke V. Using thematic analysis in psychology. Qualitative Research in Psychology. 2006;3(2):77-101.
23. Malterud K. The art and science of clinical knowledge: evidence beyond measures and numbers. Lancet. 2001;358:397-400.
24. Sherwin S, Winsby M. A relational perspective on autonomy for older adults residing in nursing homes. Health Expectations. 2011;14(2):182-90.
25. Dichter M, Sander M, Seismann-Peterson S, Köpke S. COVID-19: It is time to balance infection management and person-centered care to maintain mental health of people living in German nursing homes. International Psychogeriatrics. 2020;32(10):1157-60.
26. Naldemirci Ö, Lydahl D, Britten N, Elam M, Moore L, Wolf A. Tenacious assumptions: Exploring the tensions and variations in person-centred care. Health. 2018;22(1):54-71.
27. Wang H, Li T, Barbarino P, Gauthier S, Brodaty H, Molinuevo J, et al. Dementia care during COVID-19. The Lancet. 2020;395(10231):1190-1.
28. Verbeek H, Gerritsen D, Backhaus R, de Boer B, Koopmans R, Hamers J. Allowing visitors back in the nursing home during the COVID-19 crisis: A Dutch national study into first experiences and impact on well-being. JAMDA. 2020;21:900-4.
29. Ericson-Lidman E. Struggling between a sense of belonging and a sense of alienation: Residents' experiences of living in a residential care facilty for older people in Sweden. Nordic Journal of Nursing Research. 2019;39(3):143-51.
30. Landry M, Van den Bergh G, Hjelle K, Jalovcic D, Tuntland H. Betrayal of trust? The impact of the COVID-19 global pandemic on older persons. Journal of Applied Gerontology. 2020;39(7):687-9.
31. Armitage R, Nellums L. COVID-19 and the consequences of isolating the elderly. The Lancet Public Health. 2020;5(5):e256.
32. Hemberg J, Forsman A, Nordmyr J. One day at a time - being in the present experienced as optimal health by older adults - the existential health dimension as a health-promoting potential. Nursing and Palliative Care. 2017;2(2):1-7.

For peer review only

Standards for Reporting Qualitative Research (SRQR)

No.	Topic	Item
	**Title and abstract**	
S1	Title	Describes the nature and topic of the study. Identifying the study as qualitative and indicates the approach.
S2	Abstract	Summarises key elements of the study using the abstract format of BMJ Open; including objective, design, setting, participants, analysis, results, conclusions.
	**Introduction**	
S3	Problem formulation	Describes the significance of the phenomenon studied, and reviews previous empirical work to state the problem.
S4	Purpose or research question	Defines the purpose of the study
	**Methods**	
S5	Qualitative approach and research paradigm	Describes the study's qualitative approach
S6	Researcher characteristics and reflexivity	Describes the researchers' clinical and research experience. The researchers' reflexivity is reported on in the discussion section.
S7	Context	Describes the study setting and salient contextual factors
S8	Sampling strategy	Describes how and why research participants were selected and criteria for when no further sampling was possible.
S9	Ethical issues pertaining to human subjects	Describes approval of the local ethics review board and participant consent, under declarations. All data are anonymised, and fictional names are used to put quotations in context.
S10	Data collection methods	Describes the types of data collected, detail of data collection procedures including period of data collection.
S11	Data collection instruments and technologies	Describes the interview guide used for data collection
S12	Units of study	Describes the number and relevant characteristics of the participants included in the study.
S13	Data processing	Methods for data transcription and anonymisation/deidentification of excerpts are described in the methods section. Data management and security is described under declarations,

For peer review only

S14	Data analysis	Describes the data analysis process by which themes were identified and developed, and the extent to which the different researchers were involved in the data analysis.
S15	Techniques to enhance trustworthiness	Describes how triangulation was applied to enhance trustworthiness and credibility of data analysis.
	**Results/findings**	
S16	Synthesis and interpretation	The main findings are described in synthesised themes
S17	Links to empirical data	Anonymised quotes are used to substantiate analytical findings
	**Discussion**	
S18	Integration with prior work, implications, transferability, and contribution(s) to the field	The findings are summarised and explained in relation to earlier scholarship. The findings are also discussed in relation to generalisability and identification of the study's unique contribution to scholarship in health and social care with and for older persons.
S19	Limitations	Study limitations are described
	**Other**	
S20	Conflicts of interest	Statement that there are no conflicts of interest
S21	Funding	Sources of funding and that they had no role in any parts of the study are reported.

For peer review only

BMJ Open

Everyday life in a Swedish nursing home during the COVID-19 pandemic: a qualitative interview study with the oldest old

Journal:	*BMJ Open*
Manuscript ID	bmjopen-2020-048503.R1
Article Type:	Original research
Date Submitted by the Author:	26-Apr-2021

Complete List of Authors:	Lood, Qarin; University of Gothenburg Sahlgrenska Academy, Institute of Neuroscience and Physiology, Department of Health and Rehabilitation, Sahlgrenska Academy, Centre for Ageing and Health - AgeCap; La Trobe University School of Nursing and Midwifery<br>Haak, Maria; Kristianstad University, Research Platform for Collaboration for Health, Faculty of Health Science; Lund University Faculty of Medicine, Department of Health Sciences<br>Dahlin-Ivanoff, Synneve; University of Gothenburg Sahlgrenska Academy, Institute of Neuroscience and Physiology, Department of Psychiatry and Neurochemistry, Sahlgrenska Academy, Centre for Ageing and Health - AgeCap

**Primary Subject Heading**:	Health services research
Secondary Subject Heading:	Qualitative research
Keywords:	COVID-19, QUALITATIVE RESEARCH, PUBLIC HEALTH

SCHOLARONE™
Manuscripts

I, the Submitting Author has the right to grant and does grant on behalf of all authors of the Work (as defined in the below author licence), an exclusive licence and/or a non-exclusive licence for contributions from authors who are: i) UK Crown employees; ii) where BMJ has agreed a CC-BY licence shall apply, and/or iii) in accordance with the terms applicable for US Federal Government officers or employees acting as part of their official duties; on a worldwide, perpetual, irrevocable, royalty-free basis to BMJ Publishing Group Ltd ("BMJ") its licensees and where the relevant Journal is co-owned by BMJ to the co-owners of the Journal, to publish the Work in this journal and any other BMJ products and to exploit all rights, as set out in our licence.

The Submitting Author accepts and understands that any supply made under these terms is made by BMJ to the Submitting Author unless you are acting as an employee on behalf of your employer or a postgraduate student of an affiliated institution which is paying any applicable article publishing charge ("APC") for Open Access articles. Where the Submitting Author wishes to make the Work available on an Open Access basis (and intends to pay the relevant APC), the terms of reuse of such Open Access shall be governed by a Creative Commons licence – details of these licences and which Creative Commons licence will apply to this Work are set out in our licence referred to above.

Other than as permitted in any relevant BMJ Author's Self Archiving Policies, I confirm this Work has not been accepted for publication elsewhere, is not being considered for publication elsewhere and does not duplicate material already published. I confirm all authors consent to publication of this Work and authorise the granting of this licence.

For peer review only

Everyday life in a Swedish nursing home during the COVID-19 pandemic: a qualitative interview study with the oldest old

Qarin Lood[1,2] (0000-0002-6187-0929), Maria Haak[3,4] (0000-0002-0054-2542), Synneve Dahlin-Ivanoff[5] (0000-0002-3838-4527)
Institute of Neuroscience and Physiology, Sahlgrenska Academy, University of Gothenburg, SE-405 30 Gothenburg, Sweden. Qarin Lood senior lecturer Institute of Neuroscience and Physiology, Department of Health and Rehabilitation, Sahlgrenska Academy, Centre for Ageing and Health – AgeCap, University of Gothenburg. Maria Haak professor Research Platform for Collaboration for Health, Faculty of Health Science, Kristianstad University. Synneve Dahlin-Ivanoff professor Institute of Neuroscience and Physiology, Department of Psychiatry and Neurochemistry, Sahlgrenska Academy, Centre for Ageing and Health – AgeCap, University of Gothenburg.

Correspondence to Qarin Lood
Postal address:
Box 455
SE-405 30 Gothenburg, Sweden
Email: Qarin.lood@neuro.gu.se
Phone: 0046704942034
Fax: N/A

1. Institute of Neuroscience and Physiology, Department of Health and Rehabilitation, Sahlgrenska Academy, Centre for Ageing and Health – AgeCap, University of Gothenburg, Gothenburg, Sweden
2. School of Nursing and Midwifery, La Trobe University, Melbourne, Australia
3. Research Platform for Collaboration for Health, Faculty of Health Science, Kristianstad University, Kristianstad, Sweden
4. Department of Health Sciences, Faculty of Medicine, Lund University, Lund, Sweden.
5. Institute of Neuroscience and Physiology, Department of Psychiatry and Neurochemistry, Sahlgrenska Academy, Centre for Ageing and Health – AgeCap, University of Gothenburg, Gothenburg, Sweden

For peer review only

For peer review only - http://bmjopen.bmj.com/site/about/guidelines.xhtml

Abstract

Objective

To understand and report on the impact of the COVID-19 pandemic on the everyday lives of frail older persons living in nursing homes by exploring their experiences of how the pandemic-related restrictions had influenced them and in what way.

Design

Empirical qualitative interview study.

Setting

A publicly run nursing home in an urban area in Sweden in June 2020. The nursing home had visitor restrictions, cancelled activities and physical distancing requirements since March 2020.

Participants

A total of 10 persons 85 to 100 years, living in a Swedish nursing home during the COVID-19 pandemic were recruited through nursing home management and interviewed in June 2020 using medically approved visors and physical distancing.

Analysis

Interviews were analysed using thematic analysis, which involves familiarisation, coding and definition of themes. Transcripts were coded into data driven categories before being organised into categories that described and explained the data.

Results

The analysis resulted in the main theme "It is like living in a bubble", that describes everyday life in the nursing home during the pandemic as a world of its own in which the older persons felt both protected and isolated. This is described in four sub-themes: Living one day at a time, without fear of the virus; Feeling taken care of, Having limited freedom, and Missing out on the little extras.

Conclusions

Contributing to the growing area of COVID-19 related research, our findings provide novel insights into how pandemic-related restrictions in nursing homes represent a risk of isolating older people from the outside world, and diminishing their freedom. Put in relation to previous research, these findings could be applied beyond the pandemic, to develop research and practice that puts focus on how to support older people to decide for themselves how to spend the rest of their lives.

Keywords: COVID-19, nursing homes, social isolation, qualitative, older adults

Strengths and limitations of this study

- The qualitative approach provides novel insights into how the COVID-19 pandemic has influenced people living in nursing homes.
- The first-hand experiences described complement the medical understanding of the impact of COVID-19 on older people.
- A limitation of the study is the risk of biased analysis due to the lived experiences of the pandemic among the researchers themselves.

For peer review only

Introduction

The novel Coronavirus that is causing COVID-19 has changed pretty much everything that people do. It spreads rapidly and could cause severe and fatal infections, especially among people living in nursing homes, who are often experiencing physical frailty (1) and compromised physiological barriers (2). In Sweden, person living in nursing homes are typically living with multiple health problems and are in need of access to care staff round-the-clock (3). As per April 4 2021, a total of 16204 persons living in Swedish nursing homes had been confirmed with COVID-19, and 5446 (34%) of them had died with the disease. This constitutes 43% of the amount of people who had died with COVID-19 in Sweden at that time (4). That people in nursing homes are one of the most affected groups globally can be seen from the research produced (see for example (5-8). Out of fear of the virus, nursing home organisations around the world have made drastic changes to their services to diminish the spread of infection in line with the World Health Organization's international guidelines (9). In line with these guidelines, Swedish nursing homes implemented physical distancing and visitor restrictions, and most organised activities were cancelled homes from March 30 until October 1 2020. Although not being a ban, which is not supported by Swedish law, the visitor restrictions initiated a public debate on the risk of social isolation and the negative consequences that might have (10), and they were lifted in October 2020 based on the risk of negative consequences of isolation instigated by lengthy visitor restrictions (11). So far, research has mainly focus on family members' experiences of the restrictions (12, 13) and the impact they may have on people living in nursing homes is poorly understood, with no primary data on their experiences.

Already before the pandemic, there were reports on social isolation, limited quality of life and near endemic loneliness among people living in nursing homes (14, 15). This poses serious threats to their everyday fulfilment and sense of dignity (16-18). The pandemic tends to increase those threats (19), since restrictive measures taken to protect people residing in nursing homes from infection may have a negative impact on their wellbeing (20). Especially since social isolation is a serious public health concern as it may lead to medical illness such as cardiovascular diseases (21), and mental health problems, such as loneliness, depression and cognitive decline (22). There are also recent reports on the negative impact of social disconnection and perceived isolation on both anxiety and depression among older persons (23).

According to the Swedish government (24), a modern society and welfare state needs to improve the possibilities for all older people to participate in society, to enjoy a decent quality of life, and to experience health, meaningful activities and social participation. Research on best practice nursing home care also puts focus on activities to promote thriving and support a good quality of life (25-27), and there is evidence to suggest that quality of life is improved by participating in personally meaningful activities (28). Yet, as part of one of the risk groups for severe disease or death from COVID-19, persons living in nursing homes have faced restrictions that have resulted in no visitors, communal dining or leisure activities. The scientific community is responding rapidly to the consequences of those restrictions, but what has been left in the dark is how the persons in nursing homes themselves experience the measures taken to protect them. Therefore, this paper aimed to understand and report on the impact of the COVID-19 pandemic on the everyday lives of frail older persons living in nursing homes by exploring their experiences of how the pandemic-related restrictions had influenced them and in what way.

Methods

Seeking to understand the complexity of everyday life in a residential care home during the COVID-19 pandemic, this study had a qualitative design to explore and describe older persons' experiences. Data were collected from 10 persons in a nursing home over a two-week period in June 2020. The authors of the study are all registered occupational therapists, with research

For peer review only

expertise in gerontology, occupational science and health science. Ethical approval was obtained from The regional ethical board in Gothenburg (ref. no. 813-18).

Patient and public involvement

As part of a larger research program on user involvement in research (29), the research questions for this study were discussed in seminars with user representatives from pensioner associations in Sweden before commencement. Drawing on the findings from this study, the user representatives will also be involved in future planning of research on user involvement in research on ageing and health, together with researchers and healthcare representatives from different disciplines and professions. The findings of the study will also be presented to interested participants in dialogue with the first author, or through a short popular scientific report. They will also be presented with the choice to read the scientific report.

Study setting

Participants were recruited from a nursing home with 101 beds (27 in dementia care units), and about 85 staff members. Four of the people living in the nursing home had contracted the virus, all of them survived and were well at the time of data collection (two of them were included in the study). A few staff members had also contracted the virus and had been, or were, on sick-leave. Care staff were available for the people living there 24 hours, seven days a week. Registered nurses, physicians and allied health professionals were generally available for care and rehabilitation when needed, but during the COVID-19 pandemic, such visits were confined to acute needs. Only outdoor visits from friends and family were allowed during the study period, with a maximum of two visitors at a time. Dates and times for visits were organised by nursing home managers, and staff escorted the older persons back and forth to the outdoor area. Indoor visits were allowed for special reasons only, such as end-of-life-care or severe anxiety. Delivery of food or things from outside the nursing home had to be delivered through staff. All staff were entitled to adhere to the recommendations by the Public Health Agency of Sweden; they were strongly encouraged not go to work when feeling ill, even with mild symptoms, and to follow basal hygiene routines such as hand hygiene and disinfection of areas in contact with human beings.

For peer review only

Participants and data collection

All persons assessed by the nursing home staff as cognitively able to give informed consent and to hold a conversation for at least 15 minutes were invited to participate through receiving general written information about the study from nursing home managers. Eleven persons expressed interest and a time and place was set for the interview. All potential participants received detailed written and verbal information about the study from researchers before the interviews, and had the opportunity to ask questions about the study and their participation. Every effort was made to ensure that the choice to participate, and to what extent, was the person's own, not limited by research approaches or structures. One person withdrew their consent after the interview had been conducted.

The participants were between 85 and 100 years of age (85, 86, 89, 90, 94, 96, 98, 100, 100, and 100 years respectively). All were physically frail (30) and in need of support in activities of daily living. None of the participants had been diagnosed with dementia. Seven participants were women, eight had children and three of them had partners who were still alive. All data collected from the person who withdrew their consent were deleted and not included in the study, and no other person than the researcher who conducted the interview took part of the interview. The interviews focused on how the older persons' everyday life had been influenced by the pandemic and their increasingly frail bodies, and they lasted between 17 and 60 minutes. Medically approved visors were used in all contacts between researchers and participants and a distance of

For peer review only - http://bmjopen.bmj.com/site/about/guidelines.xhtml

at least two meters was kept at all times. All interviews were recorded digitally and transcribed before analysis.

Data analysis

Triangulation (31) was applied through constant comparative analysis to address trustworthiness. This meant that data were analysed by all three authors using the step-wise procedure for thematic analysis (32) as follows. First, all authors listened to the interviews repeatedly to become familiarised with the data. Second, initial codes that described the participants' experiences in a condensed way were generated by the second author, who had not been involved in the data collection, and discussed among all authors. Third, the initial codes were revised by the second author to search for themes in a process of listening to and reading all collected data again. Notes were taken on essential meanings and extracted data were interpreted and organised into a prospective thematic structure by the first and third author. Fourth, the prospective themes were reviewed and the authors discussed relationships between codes, themes and different levels of themes to define them and assess the validity of each prospective theme in relation to the data set as a whole. Considering internal homogeneity and external heterogeneity (33), phase four also involved a refinement of themes, and the fit of data extracts within each theme was assessed. This process was conducted in close collaboration between the authors, discrepancies were discussed and a final decision was made by the first author who was responsible for the analysis. Fifth, all data extracts were interpreted to define final themes based on the meaning and implications of the data. This process continued until it was not considered possible to conduct any further refinements of the themes. Sixth, the scientific report was written by all authors.

Results

The participants' experiences were interpreted into one main theme and four-sub-themes. The interpreted experiences are supported by quotations from a selection of participants.

It is like living in a bubble

The overarching interpretation of the participants' experiences of everyday life in the nursing home during the COVID-19 pandemic was that it was somewhat a world of its own. The pandemic-related restrictions made the participants feel safe and secure in terms of virus transmission and support in activities of daily living, but at the same time isolated from the outside world. This is described in four sub-themes: *Living one day at a time without fear of the virus, Feeling taken care of, Having limited freedom,* and *Missing out on the little extras.*

Living one day at a time, without fear of the virus

Participants handled the pandemic and their ageing bodies by living one day at a time, and when being asked if they were afraid of the virus, they expressed that there was no need to worry of getting infected since they were so old. The opportunity to live one day at a time was facilitated by the nursing home bubble in terms of protection from virus transmission and the support provided at nursing home staff that made it possible to seize the day and enjoy the activities that could proceed despite the pandemic. For instance, taking walks in the corridors, read books, do cross-words, and strive to carry out personal care activities independently to maintain one's physical and cognitive abilities. Thus, even if the ageing and physically frail body represented an insecurity as to whether pandemic-related changes to daily routines would be possible to handle, the participants experienced that they could choose to focus on, and appreciate, the small things in life and live one day at a time, rather than worrying for what might happen in the future. This was also described in terms of having no choice but to accept the situation, even if being old and frail was experienced as tough. One of the participants who had survived COVID-19 described this as:

For peer review only

"I am very tired now, because I have had this, Corona. I believe it was not too bad for me. I had a fever and they stated that it was that (Corona), but I wasn't. I had a strange feeling in my body and felt miserable, but I don't think I had the worst. Breathing is difficult anyway, cancer operation. Right now, it seems to be alright. I have other cancer as well. It has been a lot. But it's alright, you have to live one day at a time."

Feeling taken care of

Living in a nursing home during the COVID-19 pandemic contributed to a feeling that one was taken care of, both in terms of being protected from the virus and being cared for by staff. Daily routines could still be carried out, representing an important part of everyday life that was not affected by the pandemic, and the nursing home staff embodied a much-appreciated human contact in times of social isolation. Staff provided support for the participants to defy both the pandemic in terms of protection from virus transmission, and the ageing body in terms of support to maintain essential activities and daily routines. This contributed to an everyday life that, in many ways, was the same as before the pandemic. Staff also offered a sense of security in knowing that there would be someone there to care for you and provide support when needed. This is visualised by a quotation by one of the participant's response to the question about how they felt about living in the nursing home during the pandemic:

"I am very satisfied. Because of the staff, that they are so helpful. They are helpful and come in and talk with me. I have nothing to complain about… I cannot manage on my own, here I am spoiled, here I get food and the assistance that I need."

Having limited freedom

Everyday life in the shielded world of a nursing home during the pandemic involved not having the freedom to choose for oneself what to do, with whom and when. Instructions from nursing home staff and authorities were dutifully followed, even though the participants did not always agree with them. There was a perception that there simply was no option, and there were expectations on authority representatives to set a date for when the isolation would be over and everything would get back to as it was before the pandemic. The limited freedom also involved increased dependency on staff, and the participants became more inactive than before the pandemic. They experienced that their health deteriorated, both because of age and frailty, and because of the pandemic-related restrictions with few opportunities to move about, to go outside or to receive visitors without assistance from staff. Envisaging life after the pandemic, participants dreamt about moving around freely again, and regaining their strength and energy after being passive due to the restrictions, or after having COVID-19 themselves. The feeling of having limited freedom in everyday life is illustrated by a participant's answer to the question: What is the biggest difference for you since the pandemic started?

"The freedom. I am dependent you see. So, I cannot choose... Nobody is allowed to go out, nobody is allowed to come in."

Missing out on the little extras

Everyday life during the pandemic was experienced as empty and dull, without the things that were considered the little extras that provided a silver lining to everyday life. With no opportunities to go out and do errands, participants were dependent on relatives to bring them the little extra things that they could not get in any other way, e.g., fresh flowers, bakery or new clothes. Initiatives had been taken to deliver things from relatives to the participants through staff, but it was not always possible due to staff being under extra pressure during the pandemic. For this reason, participants did not want to disturb staff with things that might seem trivial, such as having a chat or to go out for a walk, and because no visitors were allowed, life was experienced as being somewhat destroyed by the pandemic-related restrictions. Everyday life in

For peer review only

the nursing home came to circle around daily routines and news on the pandemic, and the telephone came to be one of few contacts with the outside world. Although not being able to replace physical contact with friends and family, the telephone gave opportunities to think and talk about other things than the pandemic. The participants also missed doing fun and meaningful activities such as excursions or organised social and creative activities, and even if they understood why these types of activities were cancelled to minimise virus transmission, they lacked the stimulation they received from them. One woman described the difference between everyday life before and during the pandemic as:

> *"(Before Corona) there were (visitors) every day almost. And there were activities in the activity room. We had such a nice time down there, we had coffee at eleven in the joint room, we had a nice time. We sang together and had quizzes, and watched tv together, and we took walks outdoors. And we got to go to the horticultural society (gardens) and now we are not allowed to do anything."*

Discussion

Statement of principal findings

To the best of our knowledge, this study is the first to explore experiences of everyday life in a nursing home during the COVID-19 pandemic. Illustrated through the metaphor of living in a bubble, everyday life at the nursing home during the COVID-19 pandemic meant living a protected but isolated life and the findings highlight the need for nursing homes to both aim to protect and sustain physical health and capacity, and to support psychosocial aspects of life. The illumination of how frail older people in nursing homes experienced the pandemic-related restrictions present serious challenges to older people's freedom to do what they have a reason to value. Thus, by giving voice to the older persons themselves, the present findings provide insights into how they themselves would have wanted everyday life during the pandemic to be. Although not expressing a willingness to die, the participants were not afraid of contracting the virus, and they wished to decide for themselves how to spend the rest of their lives, rather than being limited by restrictions to minimise virus transmission. As Ronald Bayer (34) stated already in 2007, there are continuing tensions between individual rights and public health, and the question he poses on the extent of protection of public welfare in relation to fundamental rights of individual people is perhaps even more relevant now than ever before. As Bayer points out, authoritarian measures to protect the public may be sanctioned by the notion of common good. However, even if the pandemic situation in 2020 required drastic measures, it seems as if authorities were given perhaps too much liberty to diminish virus transmission, with sometimes dire consequences for people approaching the end of life.

Strengths and weaknesses of the study

The major strength of this study is the uniqueness of the qualitative data gathered from a sample of persons 85 years and older living in nursing homes during the COVID-19 pandemic, giving voice to a seldom heard, but largely affected group of people. The findings thus provide empirical support for what until now has been assumptions by other people than the older persons themselves (20, 35). Consequently, even if the number of participants was limited due to the restrictions applied, the first-hand experiences described provide important insights to the field of nursing home care in the light of the pandemic. Moreover, as described by Malterud (36), rigour in qualitative studies depends on reflexivity and not a specified sample size (36) and although representing a Swedish example, the similarities between restrictions implemented in nursing homes around the world increases the international value of the study. The findings complement the medical understanding of the impact of COVID-19 on frail older people with an understanding of how the pandemic-related restrictions may influence older people's everyday life and well-being. Due to the qualitative design, however, it is impossible to draw any conclusions on the impact of the restrictions or the pandemic on the physical and mental health

For peer review only - http://bmjopen.bmj.com/site/about/guidelines.xhtml

of older persons in nursing homes. Another limitation with the study is the fact that the researchers were studying experiences the pandemic, while at the same time experiencing themselves. Even if the authors strove to be aware of their own expectations and experiences throughout the analysis procedure, this may have resulted in a biased analysis. Every attempt was, however, made to assure that the authors' experiences did not overshadow the voices of the older persons participating in the study.

Other studies

Interpreted in relation to previous research on autonomy among older people in nursing homes (37), the present findings visualise a risk for measures taken to protect people in nursing homes from COVID-19 to become paternalistic. Authorities and clinicians alike may believe that they know best, and older persons' interests and experienced needs may be overridden by others. Supported by what has been described by Dichter et al. (35), the findings call for a balance between infection control and person-centred care that combines biomedical knowledge of diseases with personal understanding of individual person's desires and needs. The older persons' descriptions of everyday life in a nursing home during the COVID-19 pandemic illuminate issues in terms of caring for older people as persons, and there is a need, that goes beyond the pandemic, to implement person-centred care that can support older persons in nursing homes to both remain safe and preserve their dignity and personhood by protecting them from physical threats to their health while, at the same time, attending to their psychosocial needs. As stated by Nademirci et al. (38), person-centred care involves healthcare professionals' responsibility to meet each person's needs by personalising the care and prioritise shared decision-making (38). The COVID-19 pandemic has challenged this endeavour, and highlighted a need for infection control interventions to also provide psychosocial and mental health support (39). Deepening the understanding of the negative consequences of the pandemic on older persons' everyday lives in nursing homes, the present findings thus provide a foundation for future research to attend to health and well-being in a broader sense than strict infection control.

The present findings contribute with an understanding of how pandemic-related restrictions in nursing homes may be experienced as isolation and deprivation of freedom of choice. Throughout the pandemic, visitor restrictions have been implemented without scientific support for the effect of such measures to minimise virus transmission (40), and there is a lack on primary data on how this may impact the lives of persons living in, and visiting, nursing homes. This study thus presents a unique contribution to the growing area of research on the COVID-19 pandemic, illustrating how the pandemic-related restrictions may as great a threat to the health and wellbeing of older people in nursing homes, as the virus. As described by Landry et al. (41), public health measures to reduce transmission of COVID-19 among older persons need to attend to several dimensions of health (41), and contradictions between protection from the threats of COVID-19 infection and the risk of social isolation imposed by the pandemic need to be attended to (20, 35, 42, 43). What the present study adds to this understanding of the impact of the COVID-19 pandemic on older persons in nursing homes is the first-hand perspective of representatives from a group identified as being among the most vulnerable when it comes to COVID-19 infection. Contrary to expectations, the findings illustrate how the older persons maintained many daily routines, without fear for the virus, and that they avoided pandemic worry by living one day at a time. This has been described in previous research as a strategy applied by persons approaching the end of life (44), and could be understood as a potential for health. In the light of these findings, it seems reasonable to question whether the interventions to minimise the threat of COVID-19 in nursing homes are adequate, and whether mortality is a sufficient outcome to measure effects of such interventions.

Implications of the findings and future research

For peer review only - http://bmjopen.bmj.com/site/about/guidelines.xhtml

Giving voice to persons representing one of the most vulnerable groups in terms of COVID-19 infection, this study contributes with a unique perspective on the impact of the pandemic, and not the virus per se, on the older persons' lives. Specifically, our study contributes with a visualisation of how restrictions to minimise the risk of infection may not be the most relevant intervention during the pandemic. Contributing to the growing area of COVID-19 related research, this provides a foundation for future research and practice, calling for services that can balance protection from physical threats with psychosocial support.

Authors' contributions

QL designed and directed the project and initiated contact with the management at the nursing home. She also did the literature search and drafted the manuscript. QL and SDI collected the data, and were responsible for data analysis with input from MH. SDI also contributed with intellectual input on the manuscript draft. MH was responsible for the first steps of the analysis and contributed with intellectual input to the final interpretations as well as the manuscript draft. All authors have reviewed and agreed to the draft and final version of the paper.

Acknowledgements

First and foremost, we would like to thank the persons participating in the study, sharing their experiences of their everyday lives during these challenging times. We would also like to thank the management at the nursing home for allowing us to conduct the interviews, and the care staff for assisting us with selecting participants and for their tireless commitment to providing good care and support to the persons living in the nursing home. Finally, we thank The Swedish Research Council for Health, Working Life and Welfare (FORTE) who funded the study.

Declaration of interests

None declared

Data sharing statement

The qualitative data generated and analysed as part of the current study are not publicly available due to the information provided to the participants when obtaining their informed consent, stating that all attempts would be made to maintain confidentiality. De-identified data are, however, available upon reasonable request to enable review, and will be stored for 10 years at the University of Gothenburg. All data are covered by the Public Access to Information and Secrecy act (offentlighets- och sekretesslagen) and a confidentiality assessment (sekretessprövning) will be performed at each individual request. Permission from University of Gothenburg, the Institute of Neuroscience and Physiology, has to be obtained before data can be accessed.

Ethics statement

Ethical approval was obtained from The regional ethical board in Gothenburg (ref. no. 813-18) and all participants provided written informed consent before the interviews.

Funding

This work was supported by The Swedish Research Council for Health, Working Life and Welfare (FORTE), grant number 2018–00904.

For peer review only

References

1. Kojima G. Prevalence of frailty in nursing homes: A systematic review and meta-analysis. Journal of the American Medical Directors Association. 2015;16:940-5.
2. Stone P. Integration of infection management and palliative care in nursing homes: an understudied issue. Research in Gerontological Nursing. 2017;10:199-204.
3. Socialtjänstförordning (Social Service regulation), (2001:937).
4. The National Board on Health and Welfare (Socialstyrelsen). Statistics on COVID-19 (In Swedish) www.socialstyrelsen.se: Socialstyrelsen; 2021 [Available from: https://www.socialstyrelsen.se/en/statistics-and-data/statistics/statistics-on-covid-19/.
5. Strang P, Bergström J, Lundström S. Symptom relief is possible in elderly dying COVID-19 patients: a national register study. Journal of Palliative Medicine. 2021;24(4):514-9.
6. Montoya A, G. J, Mills J, Beal J, Chun E, D. N, et al. Partnering with local hospitals and public health to manage COVID-19 outbreaks in nursing homes. Journal of the American Geriatrics Society. 2021;69(1):30-6.
7. Villaumbrosia C, Peromingo J, Imedio J, Montiel T, Suárez L-P, Clemente I, et al. Implementation of an algorithm of cohort classification to prevent the spread of COVID-19 in nursing homes. Journal of American Medical Directors Association. 2020;21(12):1811-4.
8. Song R, Kim H-S, Yoo S-J, Lee K, Park j-H, Jang JH, et al. COVID-19 in nursing facilities: experience in Republic of Korea. Osong Public Health and Research Perspectives. 2020;11(4):164-9.
9. World Health Organization. Preventing and managing COVID-19 across long-term care services: Policy Brief Geneva: World Health Organization; 2020 [Available from: https://apps.who.int/iris/handle/10665/334020.
10. Ludvigsson J. The first eight months of Sweden's COVID-19 strategy and the key actions and actors that were involved. Acta Paediatrica. 2020;109(12):2459-71.
11. The Public Health Agency of Sweden (Folkhälsomyndigheten). Preventing transmission of infection when visiting nursing homes (In Swedish) Folkhälsomyndigheten 2020 [Available from: https://www.folkhalsomyndigheten.se/smittskydd-beredskap/utbrott/aktuella-utbrott/covid-19/information-till-varden/personal-inom-aldreomsorg/forebygga-smittspridning-vid-besok-pa-aldreboenden/.
12. Yeh T-C, Huang H-C, Yeh T-Y, Huang W-T, Huang H-C, Chang Y-M, et al. Family members' concerns about relatives in long-term care facilities: Acceptance of visiting restriction policy amid the COVID-19 pandemic. Geriatrics & Gerontology International. 2020;20(10):938-42.
13. Joost W, Kolk D, van den Besselaar J, MacNeil-Vroomen J, Buurman-van E, BM., van Rijn M. Evaluating perspectives of relatives of nursing home residents on the nursing home visiting restrictions during the COVID-19 crisis: A dutch cross-sectional survey study. Journal of the American Medical Directors Association. 2020;21(12):1746-50.e3.
14. Jakobsson U, Hallberg I. Loneliness, fear, and quality of life among elderly in Sweden: a gender perspective. Aging Clinical and Experimental Research. 2005;17(6):494-501.
15. Sundström G, Fransson E, Malmberg B, Davey A. Loneliness among older Europeans. European Journal of Ageing. 2009;6(4):267-75.
16. Edvardsson D, Petersson L, Sjögren K, Lindkvist M, Sandman P. Everyday activities for people with dementia in residential aged care: associations with person-centredness and quality of life. International journal of older people nursing. 2013.
17. O'Sullivan G, Hocking C. Positive ageing in residential care. New Zealand Journal of Occupational Therapy. 2006;53(1):17-26.
18. Vernooij-Dassen M, Faber M, Olde Rikkert M, Koopmans R, van Achterberg T, Bratt D, et al. Dementia care and labour market: the role of job satisfaction. Aging and Mental Health. 2009;13(3):383–90.

For peer review only - http://bmjopen.bmj.com/site/about/guidelines.xhtml

19. Plagg B, Engl A, Piccoliori G, Eisendle K. Prolonged social isolation of the elderly during COVID-19: Between benefit and damage. Archives of Gerontology and Geriatrics. 2020;89:104086.
20. Gordon A, Goodman C, Achterberg W, Barker R, Burns E, Hanratty B, et al. Commentary: COVID in care homes - challenges and dilemmas in healthcare delivery. Age and Ageing. 2020;49(5):701-5.
21. Tomàs J, Pinazo-Hernandis S, Oliver A, Donio-Bellegarde M, Tomàs-Aguirre F. Loneliness and social support: Differential predictive power on depression and satisfaction in senior citizens. Journal of Community Psychology. 2019;47(5):1225-34.
22. Barbosa Neves B, Sanders A, Kokanović R. "It's the worst bloody feeling in the world": Experiences of loneliness and social isolation among older people living in care homes. Journal of Aging Studies. 2019;49:74-84.
23. Santini Z, Jose P, Cornwell E, Koyanagi A, Nielsen L, Hinrichsen C, et al. Social disconnectedness, perceived isolation, and symptoms of depression and anxiety among older Americans (NSHAP): a longitudinal mediation analysis. The Lancet Public Health. 2020;5(1):E62-E70.
24. Government offices of Sweden (Regeringskansliet). Goals for social services, including elederly care (In Swedish) Government offices of Sweden; 2020 [Available from: https://www.regeringen.se/regeringens-politik/socialtjanst-inklusive-aldreomsorg/mal-for-socialtjanst-inklusive-aldreomsorg/.]
25. Bergland Å, Kirkevold M. Thriving in nursing homes in Norway: contributing aspects described by residents. International Journal of Nursing Studies. 2006;43(6):681-91.
26. Cooney A. Resident perspectives of the determinants of quality of life in residential care in Ireland. Journal of Advanced Nursing. 2009;65(5):1029-38.
27. Zingmark K, Sandman P, Norberg A. Promoting a good life among people with Alzheimer's disease. Journal of Advanced Nursing. 2002;38(1):50-8.
28. Cooney A, Murphy K, O'Shea E. Resident perspectives of the determinants of quality of life in residential care in Ireland. Journal of Advanced Nursing. 2009;65(5):1029-38.
29. Iwarsson S, Edberg A, Dahlin-Ivanoff S, Hanson E, Jönson H, Schmidt S. Understanding user involvement in research in aging and health. Gerontology and Geriatric Medicine. 2019;5(18):661.web.
30. Eklund K, Wilhelmson K, Landahl S, Dahlin-Ivanoff S. Screening for frailty among older emergency department visitors: Validation of the new FRESH-screening instrument. BMC Emergency Medicine. 2016;16(1):27.
31. Merriam S. Qualitative research: A guide to design and implementation. San Francisco, CA.: John Wiley & Sons; 2009.
32. Braun V, Clarke V. Using thematic analysis in psychology. Qualitative Research in Psychology. 2006;3(2):77-101.
33. Patton M. Qualitative evaluation and research methods, second edition. London: Sage; 1990.
34. Bayer R. The continuing tensions between individual rights and public health: Talking point on public health versus civil liberties. European Molecular Biology Organization. 2007;8(12):1099-103.
35. Dichter M, Sander M, Seismann-Peterson S, Köpke S. COVID-19: It is time to balance infection management and person-centered care to maintain mental health of people living in German nursing homes. International Psychogeriatrics. 2020;32(10):1157-60.
36. Malterud K. The art and science of clinical knowledge: evidence beyond measures and numbers. Lancet. 2001;358:397-400.
37. Sherwin S, Winsby M. A relational perspective on autonomy for older adults residing in nursing homes. Health Expectations. 2011;14(2):182-90.

For peer review only

38. Naldemirci Ö, Lydahl D, Britten N, Elam M, Moore L, Wolf A. Tenacious assumptions: Exploring the tensions and variations in person-centred care. Health. 2018;22(1):54-71.
39. Wang H, Li T, Barbarino P, Gauthier S, Brodaty H, Molinuevo J, et al. Dementia care during COVID-19. The Lancet. 2020;395(10231):1190-1.
40. Verbeek H, Gerritsen D, Backhaus R, de Boer B, Koopmans R, Hamers J. Allowing visitors back in the nursing home during the COVID-19 crisis: A Dutch national study into first experiences and impact on well-being. JAMDA. 2020;21:900-4.
41. Landry M, Van den Bergh G, Hjelle K, Jalovcic D, Tuntland H. Betrayal of trust? The impact of the COVID-19 global pandemic on older persons. Journal of Applied Gerontology. 2020;39(7):687-9.
42. Armitage R, Nellums L. COVID-19 and the consequences of isolating the elderly. The Lancet Public Health. 2020;5(5):e256.
43. Hemberg J, Forsman A, Nordmyr J. One day at a time - being in the present experienced as optimal health by older adults - the existential health dimension as a health-promoting potential. Nursing and Palliative Care. 2017;2(2):1-7.
44. Ericson-Lidman E. Struggling between a sense of belonging and a sense of alienation: Residents' experiences of living in a residential care facilty for older people in Sweden. Nordic Journal of Nursing Research. 2019;39(3):143-51.

For peer review only

For peer review only - http://bmjopen.bmj.com/site/about/guidelines.xhtml

Standards for Reporting Qualitative Research (SRQR)

No.	Topic	Item
	**Title and abstract**	
S1	Title	Describes the nature and topic of the study. Identifying the study as qualitative and indicates the approach. (Title page)
S2	Abstract	Summarises key elements of the study using the abstract format of BMJ Open; including objective, design, setting, participants, analysis, results, conclusions. (Page 2)
	**Introduction**	
S3	Problem formulation	Describes the significance of the phenomenon studied, and reviews previous empirical work to state the problem. (Page 3)
S4	Purpose or research question	Defines the purpose of the study. (Page 3)
	**Methods**	
S5	Qualitative approach and research paradigm	Describes the study's qualitative approach. (Page 3 and 4)
S6	Researcher characteristics and reflexivity	Describes the researchers' clinical and research experience. (Page 4). The researchers' reflexivity is reported on in the discussion section. (Page 8)
S7	Context	Describes the study setting and salient contextual factors. (Page 4)
S8	Sampling strategy	Describes how and why research participants were selected and criteria for when no further sampling was possible. (Page 4-5)
S9	Ethical issues pertaining to human subjects	Describes approval of the local ethics review board and participant consent, under declarations. All data are anonymised, and fictional names are used to put quotations in context. (Page 4)
S10	Data collection methods	Describes the types of data collected, detail of data collection procedures including period of data collection. (Page 4-5)
S11	Data collection instruments and technologies	Describes the interview guide used for data collection. (Page 4-5)
S12	Units of study	Describes the number and relevant characteristics of the participants included in the study. (Page 4)
S13	Data processing	Methods for data transcription and anonymisation/deidentification of excerpts are described in the methods section. (Page 5) Data management and security is

For peer review only

		described under Data sharing statement (Page 9-10)
S14	Data analysis	Describes the data analysis process by which themes were identified and developed, and the extent to which the different researchers were involved in the data analysis. (Page 5)
S15	Techniques to enhance trustworthiness	Describes how triangulation was applied to enhance trustworthiness and credibility of data analysis. (Page 5)
	**Results/findings**	
S16	Synthesis and interpretation	The main findings are described in synthesised themes. (Page 5-7)
S17	Links to empirical data	Anonymised quotes are used to substantiate analytical findings. (Page 6-7)
	**Discussion**	
S18	Integration with prior work, implications, transferability, and contribution(s) to the field	The findings are summarised and explained in relation to earlier scholarship. The findings are also discussed in relation to generalisability and identification of the study's unique contribution to scholarship in health and social care with and for older persons. (Page 7-9)
S19	Limitations	Study limitations are described. (Page 8)
	**Other**	
S20	Conflicts of interest	Statement that there are no conflicts of interest. (Page 9)
S21	Funding	Sources of funding and that they had no role in any parts of the study are reported. (Page 5)

For peer review only
